# Genomic analysis revealed a novel genotype of methicillin-susceptible *Staphylococcus aureus* isolated from a fatal sepsis case in dengue patient

**DOI:** 10.1038/s41598-021-83661-8

**Published:** 2021-03-01

**Authors:** Soo Tein Ngoi, Wen Kiong Niek, Yee Wan Lee, Sazaly AbuBakar, Cindy Shuan Ju Teh

**Affiliations:** 1 Department of Medical Microbiology, Faculty of Medicine, University of Malaya, 50603 Kuala Lumpur, Malaysia; 2 Department of Medicine, Faculty of Medicine, University of Malaya, 50603 Kuala Lumpur, Malaysia; 3 Tropical Infectious Diseases Research and Education Centre (TIDREC), University of Malaya, 50603 Kuala Lumpur, Malaysia

**Keywords:** Microbiology, Bacteriology, Clinical microbiology, Microbial genetics

## Abstract

*Staphylococcus aureus* (*S. aureus*) is an opportunistic pathogen capable of causing serious health implications in susceptible individuals once it invades the host’s protective barriers. Methicillin-susceptible *S. aureus* (MSSA) often receives lesser attention although it has been frequently associated with serious infections in human. We aim to investigate the genomic features of a highly virulent yet pan susceptible MSSA strain (coded as HS-MSSA) which caused concurrent bacteraemia in a dengue patient, ultimately resulted in sepsis death of the patient. Whole genome sequence analysis was performed. The draft genome of HS-MSSA is approximately 2.78 Mb (GC content = 32.7%) comprising of 2637 predicted coding sequences. In silico genotyping of the HS-MSSA strain revealed a novel combined genotype (t091/ST2990). The HS-MSSA carries a SaPIn1-like pathogenicity island that harbours the staphylococcal enterotoxin and enterotoxin-like genes (*sec*3 and *sel*L). The strain-specific β-lactamase (*bla*Z)-bearing plasmid region was identified in HS-MSSA. Core genome phylogeny showed that the HS-MSSA strain shared a common ancestry with the European MRSA clone. We report herein the genomic features of an MSSA lineage with novel genotype previously not reported elsewhere.

## Introduction

*Staphylococcus aureus* (*S. aureus*) is a Gram-positive round-shaped bacterium commonly found colonizing the skin and mucous membranes of humans. Approximately 15% of the human population carry *S. aureus* in the nasal cavity, specifically in the anterior nares^1^. *S. aureus* is an opportunistic pathogen capable of causing serious health implications in susceptible hosts when it enters the bloodstream or internal tissues. Potentially serious infections caused by *S. aureus* include bacteraemia, pneumonia, endocarditis, and osteomyelitis^2^. In fact, the nasal carriage of *S. aureus* has shown a strong association with the aetiology of bacteraemia in susceptible hosts^3^. Bacteraemia caused by invasive *S. aureus* often results in greater mortality rate due to its ability to secrete a wide spectrum of virulence factors that contribute to the evasion of host immune responses^4^.

The severe consequences of *S. aureus* infections are often associated with healthcare facilities due to the breaching of the skin barrier with surgical devices or implants, although it is not uncommon to have community acquired infections^5^. Antimicrobial resistance (AMR) among the healthcare-associated *S. aureus* infections has been an important global public health issue. Since the emergence of methicillin-resistant *S. aureus* (MRSA) in the 1960s, this organism has been notorious for its ability to cause severe forms of infection in hospital settings and has eventually spread to the community after 30 years of evolution, most probably due to the transfer of the AMR gene cassette SCC*mec* to local methicillin-susceptible lineages^6^. This evolutionary theory for the emergence of MRSA lineage has recently been strengthened by whole genome sequence analysis^7^.

Despite receiving less attention compared to its drug-resistant counterpart, the methicillin-susceptible *S. aureus* (MSSA) infection has persisted in both healthcare settings and the community, amidst the increasing global prevalence of MRSA^8^. In fact, a slow epidemic shift was observed in *S. aureus* infections worldwide during the past two decades. In the United States (US), multiple clinical studies have recorded an increase in the prevalence of MSSA and a simultaneous decrease in MRSA incidences since 2005^9,10^. Recent surveillance in the US has seen an increase in bloodstream infections caused by community acquired MSSA from 2012 to 2017^11^. A similar observation was recorded in Europe, whereby a decrease in MRSA bacteraemia was observed since 2005, accompanied by a continuous increase in MSSA bacteraemia^12^.

Genotyping studies from different geographical regions often revealed a greater genetic diversity among the MSSA populations compared to the MRSA^13–15^. More often than not, invasive MSSA causes greater illness severity in patients suffering from bacteraemia due to its ability to express multiple virulence factors^16,17^. Community-onset MSSA bacteraemia has documented a higher mortality rate compared to that of MRSA in many regions^18^. These observations showed that while much attention has been directed to MRSA infections in recent years, the often neglected MSSA infections have caused a greater and increasing disease burden worldwide.

The ability of the diverse MSSA lineages to express a multitude of virulence genes and the possibility of developing multi-drug resistance phenotypes due to horizontal gene transfer have posed a great risk to public health. Nonetheless, the genetic drives that are responsible for the emergence and persistence of such virulent MSSA lineages remain obscured. In this study, we aimed to investigate the genomic features of an invasive MSSA strain which caused a fatal sepsis case in human. The patient was initially diagnosed with dengue fever upon admission to the hospital, but later developed pneumonia and severe sepsis rapidly and succumbed to multiple organs failure within 24 h post-admission.

## Materials and methods

### Bacterial strain

The community acquired MSSA strain (coded as HS-MSSA) was isolated from the blood culture of a patient in 2017. The clinical presentations as well as the AMR and virulence characteristics related to the infection have been previously described^19^.

### Whole genome sequencing, assembly, and annotation

The genomic DNA of the HS-MSSA strain was extracted using DNeasy Blood & Tissue Kit (Qiagen, Hilden, Germany) and the quality of the extracted genomic DNA was evaluated based on spectrophotometric measurements^20^. The HS-MSSA genomic DNA was subsequently subjected to next-generation sequencing via Illumina Genome Analyzer_IIx_ (GA2x, pipeline version 1.80) by a commercial sequencing vendor. Paired-end 2 × 150 bp reads were generated using Illumina chemistry. The quality of the sequence reads was assessed and assembled de novo using the CLC Genomics Workbench version 5.1 (CLC Bio, Aarhus, Denmark) with default parameters. Contigs with an average coverage of 30 and above were selected to represent the bacterial draft genome for further analyses. Reordering of the HS-MSSA contigs was achieved via genomic mapping with the reference genome *S. aureus* subspecies *aureus* NCTC 8325 (National Center for Biotechnology Information (NCBI) GenBank accession number: NC007795) by using Mauve software version 2.4.0^21^. The subset of the contigs that were mapped to the complete chromosome of the *S. aureus* NCTC 8325 was identified as the chromosomal region of the HS-MSSA strain. Genomic annotation was performed using the webserver Rapid Annotations using Subsystems Technology (RAST; http://rast.nmpdr.org/)^22^. The viewing and preliminary sequence-based comparison between the annotated HS-MSSA and reference genomes were done using SEED Viewer version 2.0 (http://pubseed.theseed.org/)^23^, and further validated by using Mauve.

### Whole genome sequence analyses

The chromosomal region of the HS-MSSA was subjected to a microbial genome search using the NCBI Basic Local Alignment Search Tool (BLAST; https://blast.ncbi.nlm.nih.gov/Blast.cgi) to identify genetically similar *S. aureus* strains (taxonomy ID: 46170) from the complete and draft genomes database curated in the NCBI GenBank. The BLAST search was performed using the discontinuous megablast algorithm with default parameters. Multiple genomes alignment and visualization were performed using Mauve and BLAST Ring Image Generator (BRIG) software^24^. The genotype of the HS-MSSA strain was inferred *in-silico* by using web-based servers. The conventional seven-gene multi-locus sequence typing (MLST), whole genome MLST (wgMLST), and ribosomal MLST (rMLST) predictions made use of the *S. aureus* database in PubMLST (https://pubmlst.org/saureus/) sited at the University of Oxford^25^. The online analysis tools available from the Center for Genomic Epidemiology (CGE) server (http://www.genomicepidemiology.org/) were used to predict the staphylococcal protein A (*spa*) type, presence of SCC*mec* elements, plasmid elements, and pathogenicity genes in the HS-MSSA genome^26–29^. Phage-associated genes and genomic regions in the HS-MSSA genome was identified using PHAge Search Tool Enhanced Release server (PHASTER; http://phaster.ca/)^30,31^. The genomic islands were predicted using IslandViewer 4 (http://www.pathogenomics.sfu.ca/islandviewer/) ^32^. All genomic predictions were completed using default parameters, validated by cross-examination with RAST-annotation output, and manually interrogated using NCBI BLAST nucleotide search tool.

### AMR genes prediction

The online tool, Resistance Genes Identifier version 5.1.0 (RGI; https://card.mcmaster.ca/analyze/rgi) was used to identify AMR genes in the assembled HS-MSSA genome^33^. The AMR genes selection criteria were set to perfect (100% identity) and strict (> 95% identity) hits only to the curated reference sequences in the Comprehensive Antibiotic Resistance Database (CARD; https://card.mcmaster.ca/). Prediction of partial genes was accepted as the HS-MSSA genome examined in this study resembles an incomplete genome. The RGI predictions were cross-checked with ResFinder version 3.2 (https://cge.cbs.dtu.dk/services/ResFinder/). AMR genes prediction results obtained using default parameters^34^. All predicted AMR genes were validated by cross-examination with RAST-annotation output and manually interrogated using NCBI BLAST.

### Core genome single-nucleotide polymorphisms-based phylogenetic analysis

The online tool, Reference sequence Alignment based Phylogeny builder version 1.12 (REALPHY; https://realphy.unibas.ch/realphy/) was used to identify relevant single nucleotide polymorphism (SNP) sites for core genome phylogenetic analysis^35^. Complete and draft genomes of 20 global *S. aureus* strains were selected for core genome SNP-based (cgSNP) phylogenetic analysis based on considerations including high genetic identity (from microbial genome BLAST analysis), geographical distribution, type of organisms (MSSA and MRSA), and MLST clonal complexes. The selected *S. aureus* genomes were retrieved from the NCBI GenBank and PubMLST databases and the strains information is tabulated and appended as Supplementary Table S1. SNP calling was performed using default input parameters and *S. aureus* NCTC 8325 was used as the reference genome for multiple alignments. The multiple genomes sequence alignment generated by the REALPHY tool was subsequently used to construct a phylogenetic tree. An unrooted tree was inferred via the approximate maximum likelihood (ML) method using FastTree2 software^36^, and visualized using FigTree version 1.4.3 (https://github.com/rambaut/figtree/releases)^37^. 100 bootstrap replicates were performed to support the phylogenetic inferences made in this study for practical reasons. A bootstrap analysis with 100–500 replicates is sufficient to produce qualitatively comparable support values with more than 99.5% correlation with the reference values in ML trees with higher numbers of bootstrap replicates^38^.

### 7-loci MLST-based phylogenetic analysis

The concatenated allelic sequences of the seven housekeeping genes (*arc*C, *aro*E, *glp*F, *gmk*, *pta*, *tpi*, and *yqi*L) for the central STs of the major clonal complexes (CC1, CC5, CC8, CC15, and CC97) and other frequently encountered clonal complexes (CC22, CC30, CC45, and CC93) were used to construct a phylogenetic tree. Multiple alignments of the concatenated sequences were performed by Molecular Evolutionary Genetics Analysis (MEGA) X^39^. Next, the aligned sequences were subjected to a statistical selection of best-fit phylogenetic model using jModelTest version 2.1.6^40^. An unrooted tree was subsequently inferred via the ML method using MEGA X software.

## Results and discussion

### Genomic features of HS-MSSA strain

The size of the HS-MSSA draft genome is approximately 2.78 Mb (GC content = 32.7%), of which 2,740,810 bp belongs to the genomic region that corresponds to the chromosome of *S. aureus* NCTC 8325. A total of 2,637 coding sequences (CDSs) and 65 RNAs were predicted in the HS-MSSA draft genome. The general features of the HS-MSSA draft genome are summarized in Table 1. Majority of the total predicted CDSs are shared between HS-MSSA and the reference genome NCTC 8325 (n = 2,415), with a percentage identity that ranged from 31 to 100% (median = 99.75%). A total of 222 CDSs were found unique to the HS-MSSA genome, mainly encoding for hypothetical proteins, phage proteins, mobile genetic elements, efflux pumps, virulence and resistance factors. This Whole Genome Shotgun project has been deposited at the NCBI GenBank under the accession number VCMW00000000. The version described in this paper is version VCMW01000000.

**Table 1:** General features of the HS-MSSA draft genome.

	Whole genome	Chromosomal region	Other contigs
Genome size (bp)	2,776,811	2,740,810	36,001
Number of contigs	52	37	15
GC content (%)	32.7	32.7	34
N50 (bp)	109,723	106,828	19,763
Number of subsystems	289	286	5
Number of coding sequences (CDSs)	2637	2606	31
Number of unique CDSs^a^	222	195	27
Number of RNAs	65	60	5

^a^Identified based on sequence comparison with the NCTC8325 reference genome. Unique CDSs are present only in the HS-MSSA draft genome.

### Genomic comparison revealed a great genetic resemblance between HS-MSSA strain and MRSA clones

**Figure 1:**
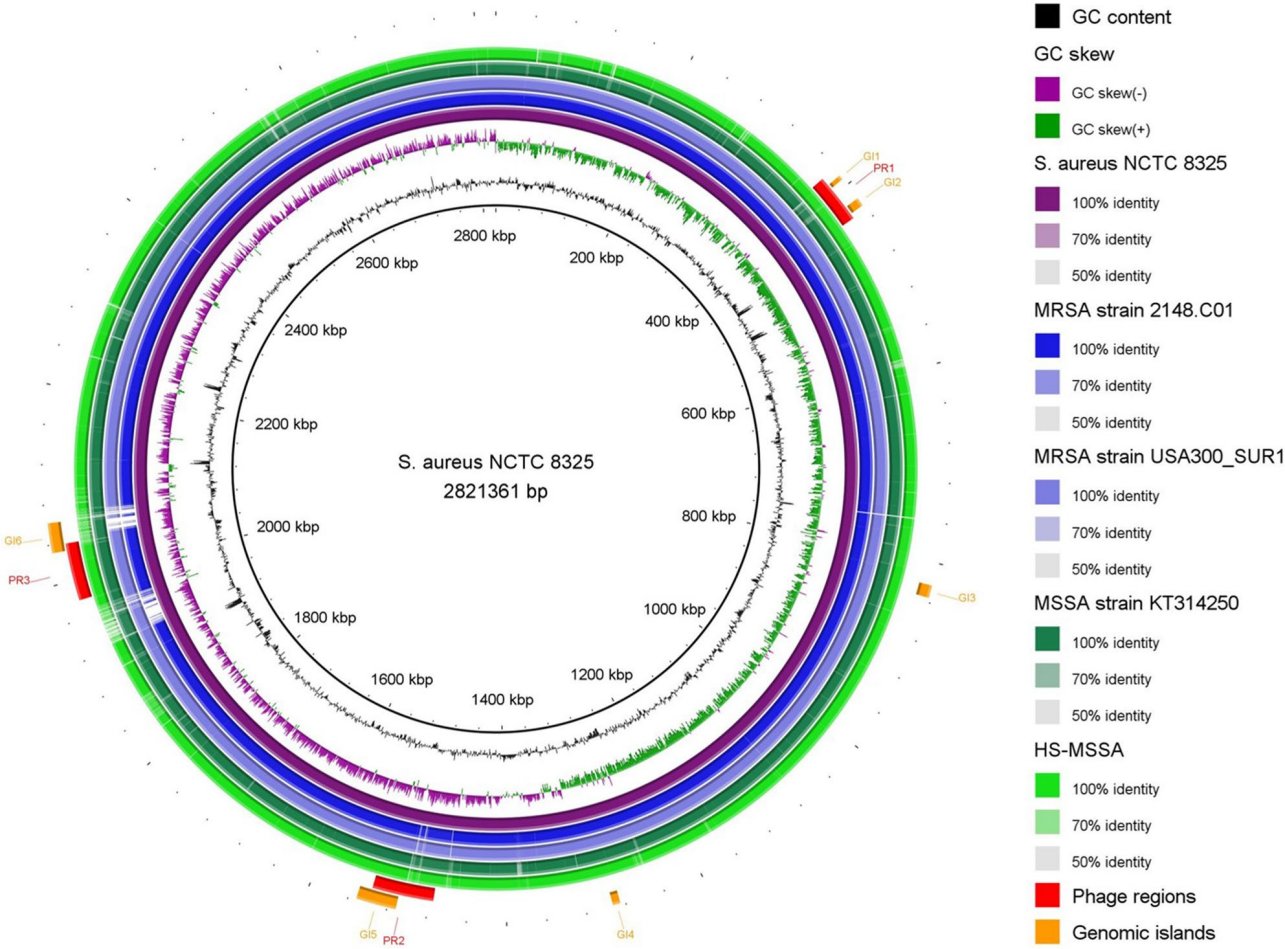
Genome map of the representative MRSA and MSSA strains. The genome map was generated using BLAST Ring Image Generator (BRIG)^24^. The genomes of two MSSA strains isolated in Malaysia (HS-MSSA and KT/314,250) and two community acquired MRSA clones (2148.C01 and USA300_SUR1 isolated in the North and South America respectively) were mapped to the reference genome *S. aureus* NCTC 8325. From the inner to the outermost ring, the first (innermost) ring shows the genome size in kbp, followed by GC skew (purple and green), GC content (black), *S. aureus* NCTC 8325 (purple), MRSA strain 2148.C01 (dark blue), MRSA strain USA300_SUR1 (light blue), MSSA strain KT/314,250 (dark green), and HS-MSSA strain (light green). The predicted phage regions (red) and genomic islands (orange) in the HS-MSSA genome are shown in arcs.

Out of the total 52 contigs generated from de novo assembly, 37 of these are associated with *S. aureus* chromosomal regions. Microbial genome BLAST analysis showed that the HS-MSSA chromosome shares greatest genetic homology (97% query coverage with 99.25% nucleotide identity at E value = 0) with the MRSA strains isolated in the United States of America (USA). More than half of the MRSA strains that are genetically similar to the HS-MSSA were obtained from a clonal cluster of community acquired MRSA that caused skin and soft tissue infections (SSTIs) in a military base in the USA^41^. Similar genetic homology was observed between the MRSA USA300 clones and HS-MSSA, albeit at slightly lower scores. The two USA300 clones that showed greatest genetic homology with HS-MSSA are USA300_TCH1516 (NC_010079) and USA300_SUR1 (NZ_CP009423), both being community acquired MRSA strains isolated from USA and Republic of Suriname in South America respectively. A circular genome map was constructed to illustrate the genetic resemblance of HS-MSSA to the representative MRSA clones from the North and South America (MRSA strains 2148.C01 (NZ_CP017094) and USA300_SUR1), and an MSSA strain KT/314250 (NZ_AOCP00000000) isolated in Malaysia (Fig. 1). The HS-MSSA strain was seen sharing more common regions of genetic variation with the MRSA clones than with the local MSSA strain at different sites on the genome.

The great genomic resemblance between the HS-MSSA strain and the MRSA clones from the American continent indicates the possible common ancestry between these lineages, despite the wide geographical distance where the strains were originated. The MRSA clones originated from the USA that showed high genetic homology with HS-MSSA were all community acquired, capable of causing SSTIs and sepsis, and were largely resistant to chlorhexidine^41–44^. The South American MRSA strain (USA300_SUR1) that caused an outbreak of SSTIs shared common MLST type (ST8) with the community acquired USA300 clone from Texas, USA^43^. The complete genome analysis revealed that the South American outbreak strains were genetic variants of the North American USA300 clone^45^. Previous genomic studies have proven that USA300 clones are clonally related to the European nosocomial MRSA strain COL (NC_002951) emerged from a methicillin-susceptible ancestral strain via the acquisition of methicillin resistance determinant following the introduction of methicillin in 1959^43,46^. Therefore, the MRSA clones USA300 and COL are most likely sharing a common MSSA ancestry but evolved separately through different MRSA lineages^47^. Accordingly, the close genomic resemblance of the HS-MSSA strain to these MRSA clones suggests that this MSSA lineage in Malaysia could have been the less evolved (with reference to AMR) descendant of the common MSSA ancestry with the MRSA clones in the USA and Europe. This inference was made on the basis that chromosomal recombination occurs in *S. aureus* at low frequencies, except for regions associated with virulence and pathogenicity^48^. The descendants of this MSSA ancestral strain have spread widely over different continents and persisted in the human populations over the years, causing community acquired SSTIs and invasive infections in the susceptible hosts.

### Presence of strain-specific blaZ-bearing plasmid in HS-MSSA

Detailed examination of the 15 contigs that were not included in the HS-MSSA chromosomal region revealed a 19,763 bp region (contig-33) identified as a plasmid-associated genomic region. BLAST analysis of contig-33 revealed the closest plasmid homolog as pl1_M2024 (CP047022), at 100% query coverage with 99% nucleotide identity (E-value = 0). The pl1_M2024 is a complete plasmid retrieved from the nosocomial MRSA clone with *spa* type t304 and MLST type ST6 (clone t304/ST6) which caused recurrent outbreaks in a hospital in Denmark since 2011^49^. This MRSA clone t304/ST6 was detected and predominated among the MRSA strains isolated in a teaching hospital in Oman within the same period^50^. However, this clone remains rarely reported outside these two geographical regions. The finding of similar plasmids in the HS-MSSA strain in Malaysia and MRSA clone in European region suggests possible horizontal gene transfer event, which could have occurred due to chance events after the divergence from the common MSSA ancestral strain. One other hypothesis is that the HS-MSSA strain could have evolved from an MSSA lineage carrying this plasmid, of which some of its descendants might have diverged to another evolutionary path via the acquisition of SCC*mec* gene cassette. The latter hypothesis is more plausible as this plasmid carries the penicillin-resistant *bla*Z gene. Based on an earlier study using *Staphylococcus* species mainly from the bovine origin, the authors demonstrated that plasmid-borne *bla*Z is often species-specific and lack conjugative transfer mechanisms, thus making horizontal gene transfer an extremely rare event^51^. Therefore, the presence of this *bla*Z-bearing plasmid in the HS-MSSA strain supports our inference on the common ancestry of this local MSSA with the MRSA clones in European regions.

### In silico genotyping of HS-MSSA revealed novel S. aureus genotype t091/ST2990

In silico MLST analyses showed that HS-MSSA belongs to the sequence type ST2990, with its identity confirmed as *S. aureus* by rMLST. The 7-loci allelic profile for HS-MSSA is 1-1-1-1-330-1-10, arranged in the order *arc*C*-aro*E*-glp*F*-gmk-pta-tpi-yqi*L. All loci MLST (wgMLST) results showed 1358 exact matches to sequence type ST2990 (Supplementary Table S2). This sequence type (ST) belongs to MSLT clonal complex CC1. The *S. aureus* strain 128A deposited in the database (PubMLST ID: 5521) shares the same sequence type as HS-MSSA and was also isolated in Malaysia, earlier in 2014. *S. aureus* 128A was isolated from the infected wound of an elderly diabetic patient (66 years old) examined in a tertiary hospital in the capital Kuala Lumpur. The strain was found susceptible to oxacillin, methicillin, and vancomycin upon investigation. The *S. aureus* 128A was confirmed as community acquired MSSA which causes wound infection. The *spa* type of HS-MSSA is t091 (*spa* repeat succession 07-23-21-17-34-12-23-02-12-23). Therefore, the combined genotype of the HS-MSSA strain is noted as t091/ST2990 (*spa* type/MLST type).

Similar to the *S. aureus* strain 128A which shares the identical sequence type (ST2990), the HS-MSSA strain examined in this study was isolated from a diabetic patient who was admitted to the hospital due to dengue fever^19^. However, instead of causing SSTI (infected diabetic wound) as strain 128A did, the HS-MSSA was more invasive and caused a bloodstream infection that resulted in severe sepsis. The common STs of *S. aureus* in Malaysia, especially the most studied MRSA strains, mainly comprised of the pandemic clone ST239, with ST22 clone being increasingly documented in the past decade^52–54^. Similar to the global epidemiology of MSSA, the few studies that examined the MSSA genotypes in Malaysia documented a higher genetic diversity compared to the MRSA populations in this region^55–57^. MRSA with *spa* type t091 has been reported in Malaysia, but *S. aureus* strain with genotype t091/ST2990 has not been previously documented^58^. Nonetheless, the isolation of ST2990 strains from different geographical regions suggests that this MSSA genotype may already be widely disseminated in Malaysia. Although *S. aureus* of CC1 complex (including ST1, ST188, ST2990, etc.) does not predominate in Malaysia, the persistence of these genotypes throughout the years may indicate their evolutionary success^55,57,58^.

### Absence of staphylococcal chromosome cassette mec elements throughout the evolutionary process of HS-MSSA

The staphylococcal chromosome cassette *mec* (SCC*mec*) elements were not detected in the HS-MSSA genome based on SCC*mec*Finder analysis at 90% identity threshold and a minimum length of 60%. The absence of SCC*mec* elements in the HS-MSSA genome is reflected in the methicillin-susceptible phenotype of this strain. The SCC*mec* genomic cassette is a mobile genomic region with two important gene complexes, *mec*-gene complex encoding methicillin resistance and cassette chromosome recombinase (*ccr*)-gene complex encoding site-specific recombinase(s) for the mobility of this DNA region^59^. The presence of SCC*mec* region in the bacterial chromosome is a signature of MRSA strains and the acquisition of which results in the emergence of new MRSA lineage from an ancestral MSSA strain.

While the acquisition of SCC*mec* elements provides a selective advantage to *S. aureus*, it is not uncommon for it to spontaneously excised from the MRSA in human hosts^60^. Partial excision is sufficient to result in methicillin-susceptible phenotype, and most often such MSSA strains showed great genetic homology to its MRSA counterpart, although less prevalent^60,61^. Given the genomic resemblance of the HS-MSSA strain to the global MRSA clones, we decided to investigate whether such an excision event has occurred in this local MSSA strain. In order to identify possible remnants of SCC*mec* cassette or its homologs in the genome, the database search was repeated at 60% identity threshold with 30% minimum query cover length. The search result remains the same, i.e. intact or partial *mec* genes and the whole cassette was not detected, but a 1.5 kb DNA region associated with the insertion sequence-like IS1272 in *SCCmec* type I was detected in contig-4, albeit with considerable genetic variation (94% query coverage and 82% nucleotide identity). BLAST analysis of this 1.5 kb region revealed the presence of a pseudogene of IS1182 family transposase in *S. aureus* (CP039848; 98% query coverage, 99% nucleotide identity, E value = 0). In short, none of the SCC*mec* elements was detected except for the 1.5 kb DNA region homologous to IS1272 commonly found in the *mec*-gene complex in MRSA that may also present infrequently in MSSA^62^.

### Pathogenicity analysis of HS-MSSA revealed limited antimicrobial resistance-conferring genes but multiple virulence factors

The HS-MSSA strain was found susceptible to amoxicillin-clavulanate, cefoxitin, ceftriaxone, ciprofloxacin, clindamycin, cloxacillin, erythromycin, fusidic acid, gentamicin, penicillin-G, piperacillin-tazobactam, rifampicin, and sulfamethoxazole-trimethoprim^19^. Genomic sequence analysis revealed a low number of AMR-conferring genes compared to virulence genes, and a majority of which are involved in bacterial efflux systems (Table 2). Partial genes were not identified in the HS-MSSA genome. The pan-susceptibility of the HS-MSSA strain to antimicrobial agents is reflected in its genomic composition. Although multiple efflux pumps and its regulatory systems involve in antimicrobial agents and biocides resistance are present in the HS-MSSA genome, it is not surprising to observe a susceptible phenotype in the strain. Similar to the Gram-negative bacteria, efflux pumps in Gram-positive organisms often confer only low-level resistance and are only effective when overexpressed due to accumulation of mutations in response to environmental stresses^63^. A recent study has reported an interesting phenomenon, whereby the prevalence of certain efflux pump genes was found to be associated with the genotypes of *S. aureus* ^64^. The presence of *nor*A and *mep*A genes in the HS-MSSA strain is typical of *S. aureus* lineages in the Asian region, while *nor*B or *mde*A is common among European *S. aureus* ^64^. Unlike plasmid-borne genes, these efflux pump genes are chromosomally located hence is fairly conserved within the *S. aureus* lineages. The close genetic proximity of the HS-MSSA strain carrying *nor*A and *mep*A genes to the European MRSA lineages suggests that the divergence of these two *S. aureus* lineages might have occurred early in the evolutionary timeline, followed by local adaptations in the different geographical regions.

**Table 2:** Antimicrobial resistance-conferring genes identified in the HS-MSSA genome.

AMR gene	Contig	Region position (bp)	SNP	Genomic region	Drug class	AMR gene family
arlS	7	73,606–74,961	–	Chromosome	Fluoroquinolone antibiotic; acridine dye	Major facilitator superfamily (MFS) antibiotic efflux pump
arlR	7	74,958–75,617	–	Chromosome	Fluoroquinolone antibiotic; acridine dye	Major facilitator superfamily (MFS) antibiotic efflux pump
norA	16	13,813–14,979	–	Chromosome	Fluoroquinolone antibiotic	Major facilitator superfamily (MFS) antibiotic efflux pump
mgrA	16	22,630–23,073	–	Chromosome	Fluoroquinolone antibiotic; cephalosporin; penam; tetracycline antibiotic; peptide antibiotic; acridine dye	ATP-binding cassette (ABC) antibiotic efflux pump; major facilitator superfamily (MFS) antibiotic efflux pump
lmrS	21	40,760–42,205	–	Chromosome	Macrolide antibiotic; aminoglycoside antibiotic; oxazolidinone antibiotic; diaminopyrimidine antibiotic; phenicol antibiotic	Major facilitator superfamily (MFS) antibiotic efflux pump
mepR	32	38,133–38,552	–	Chromosome	Glycylcycline; tetracycline antibiotic	Multidrug and toxic compound extrusion (MATE) transporter
mepA	32	38,659–40,014	–	Chromosome	Glycylcycline; tetracycline antibiotic	Multidrug and toxic compound extrusion (MATE) transporter
blaZ	33	4979–5824	–	Plasmid	Penam	blaZ beta-lactamase
tet(38)	43	1800–3158	–	Chromosome	Tetracycline antibiotic	Major facilitator superfamily (MFS) antibiotic efflux pump
GlpT variant	32	40,641–41,999	A100V	Chromosome	Fosfomycin	GlpT

The pathogenicity genes analysis showed that the HS-MSSA harbours 536 CDSs which matched to known *S. aureus* pathogenicity families (with 59% uncharacterized or hypothetical proteins). Cross-examination with virulence genes analysis showed that the HS-MSSA strain carries multiple genes encoding for bacterial adherence, enzyme production, host immune evasion, various prominent staphylococcal toxins, a type VII secretion system, and intact regulatory operons for virulence expression (global accessory gene regulator operon, *agr*ABCD; staphylococcal accessory regulator, *sar*). The virulence analysis of the HS-MSSA strain is reported in detail in our earlier publication^19^ (Supplementary Table S3). Majority of the prominent toxin-producing genes are found associated with the highly variable genomic regions in the HS-MSSA genome. The HS-MSSA resembles a community acquired, highly virulent but pan-susceptible *S. aureus* strain that was capable of causing an invasive bloodstream infection in the human host that resulted in fatal sepsis. It is a common notion that the MSSA lineages that cause human infections often harbour a wider array of virulence genes compared to the MRSA^16^. The combined effect of multiple virulent factors present in the MSSA genomes has resulted in greater severity of infection, especially one that causes invasive infection^17^. This notion is experimentally proven in a study that examined the transcriptional changes in an MRSA strain when exposed to different antibiotics^65^. In this study, Choe and colleagues proved that the expression of the β-lactam resistance (Bla/Mec system) in the MRSA represses the transcription of the *agr* operon, thus halting the production of the virulence factors that are regulated by this operon. Therefore, the ability of the HS-MSSA to cause severe sepsis in the human host could be related to the lack of SCC*mec* genes cassette in its genome, thus the expression of its multiple virulence factors was not impeded.

### HS-MSSA harbours multiple genomic islands associated with pathogenicity and prophages

**Figure 2:**
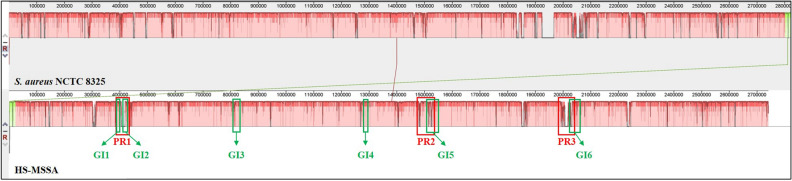
The genomic alignment of HS-MSSA chromosome against the reference genome *S. aureus* NCTC 8325. The similarity plot was generated using Mauve version 2.4.0^21^. Genomic regions with a low percentage of similarity are seen as gaps in the similarity plot of the respective genomes. The relative positions of the phage-associated regions (denoted as PR1 to PR3) and the genomic islands (denoted as GI1 to GI6) on the HS-MSSA chromosome are highlighted in red and green boxes respectively.

Three prophage regions were identified in the HS-MSSA chromosome. Phage region 1 and 3 (PR1 and PR3) are incomplete prophages, while phage region 2 (PR2) is an intact prophage spanning a 64.1 kb genomic region (Table 3). The PR1 was identified on contig-13 of the HS-MSSA genome which mainly comprised of staphylococcal toxin-production genes that encode for exotoxins and the superantigen enterotoxins (SEC3 and SElL). Genome mapping showed that this genomic region is not present in the staphylococcal reference genome NCTC 8325, thus resembling a genomic insertion (Fig. 2). The intact prophage PR2 was located on contig-4 and found associated with genes encoding for staphylococcal Panton-Valentine leukocidin (*luk*FS-PV). When mapped to the reference genome *S. aureus* NCTC 8325, the PR2 showed great genetic similarity with a 72 kb prophage on NCTC 8325. The PR3 was identified on contig-11, closely associated with genes encoding for β-hemolysin (*hlb*), cytolytic pore-forming protein (*luk*GH), extracellular adherence protein (*eap*), and staphylococcal complement inhibitor (*scn*). A total of six genomic islands (GI1-GI6) were identified in the HS-MSSA chromosome by using *S. aureus* NCTC 8325 as the reference (Fig. 2). The genomic size, contigs involved, and the types of genes identified in the genomic islands (GIs) are listed in Table 4. Generally, the regions encoding GI1-GI2, GI5, and GI6 correspond with the phage regions PR1, PR2, and PR3, respectively. The genomic region that spanned GI1 and GI2 resembles a staphylococcal pathogenicity island (SaPI) harbouring the enterotoxin-encoding genes *sec*3 and *sel*L similar to the SaPIn1 in the MRSA strain N315 (BA000018, previously AP003129)^66,67^. An array of staphylococcal virulence genes mainly associated with bacterial adherence and stress response were found in GI4, while GI5 consisted of mainly hypothetical proteins.

**Table 3:** Phage-associated regions in the chromosome of the HS-MSSA strain.

Region	Region length (kb)	Region position^a^ (bp)	Ordered contig numbers	GC %	PHASTER annotation	Phage identity^b^ (%)	PHASTER prediction^c^
PR1	51.5	379,258–430,819	13	31.6	PHAGE_Staphy_PT1028_NC_007045	9	incomplete
PR2	64.1	1,475,209–1,539,349	15, 4	32.5	PHAGE_Staphy_phi2958PVL_NC_011344	40	intact
PR3	60.1	1,978,784–2,038,945	11	31.8	PHAGE_Staphy_phiN315_NC_004740	52	incomplete

^a^Identified prophages were over multiple contigs that were concatenated for PHASTER analysis.

^b^Phage identity was calculated based on the percentage of phage-associated genes over the total protein-coding sequences identified in the phage region.

^c^A prediction of whether the phage region contains genes necessary for lysogeny.

**Table 4:** Genomic islands identified in the HS-MSSA chromosome.

Region	Region position^a^ (bp)	Region length (bp)	Ordered contig numbers	Gene products
GI1	388,058–392,685	4627	13	Ribosomal proteins; DNA-binding proteins; SaPI proteins
GI2	416,923–425,593	8670	13	SaPI proteins; phage proteins; enterotoxins (SEC3, SElL)
GI3	822,496–835,323	12,827	30, 2	Fibrinogen-binding protein (ClfA); extracellular and plasma-binding protein; staphylococcal adhesins; thermonuclease; cold-shock protein
GI4	1,285,799–1,292,852	7053	27	Hypothetical proteins
GI5	1,511,627–1,551,821	40,194	4	Phage proteins
GI6	2,031,470–2,061,814	30,344	11	Phage proteins

^a^Identified genomic islands were over multiple contigs that were concatenated for IslandViewer4 analysis.

Majority of the genomic islands identified in the HS-MSSA genome are associated with bacterial virulence and pathogenicity. Genomic islands are specific chromosomal DNA regions that include genes conferring a survival advantage to the bacterial host, such as virulence and resistance^59^. The association of the genomic islands, prophages, and SaPI observed in the HS-MSSA genome is not uncommon as these genetic entities are often interrelated in *S. aureus*. Pathogenicity islands are genomic islands that carry virulence factors and are mobilized by helper phages^66^. Moreover, prophages and pathogenicity islands are mobile elements in the staphylococcal chromosome that constitutes the genetic diversity among different *S. aureus* lineages. The prevalence of specific staphylococcal prophages was shown associated with the clonal background of *S. aureus*, i.e. different lineages may harbour different types of prophage^68^. Meanwhile, the SaPIs actively involved in the pathogenic evolution of *S. aureus* via the lateral transfer of virulence genes^69^, hence a greater genetic homology in SaPIs may indicate a closer phylogenetic relationship. Therefore, the sharing of genetically similar SaPIs between HS-MSSA and MRSA N315, and common phage regions between HS-MSSA and NCTC 8325 suggest a clonal relationship, or more probably, the sharing of a recent common ancestor that acquired the common phage or SaPI regions prior to evolutionary divergence.

### Phylogenetic analysis revealed common ancestry of HS-MSSA with MRSA clones originated from European regions

Representative *S. aureus* complete genomes were selected for cgSNP-based phylogenetic analysis to elucidate the probable evolutionary origin of HS-MSSA strain. The reference genomes were selected based on greatest nucleotide identity in BLAST analysis (MRSA strain 2148.C01 and USA300 clones), sharing of similar mobile genomic regions (M2024, NCTC 8325 and N315), same geographical origin (KT/314250), same clonal complex (MSSA476), and representative MSSA and MRSA genomes (MSSA476 and COL) from European region (potential common ancestry). The HS-MSSA genome showed ≥ 94% genome coverage and ≥ 99.16% nucleotide identity (E value = 0) with all selected *S. aureus* genomes. The ST22 reference genome HO 5096 0412 (NC_017763) from a clonal complex (CC22) less closely related to other *S. aureus* strains was used as an outgroup in constructing the ML tree.

**Figure 3:**
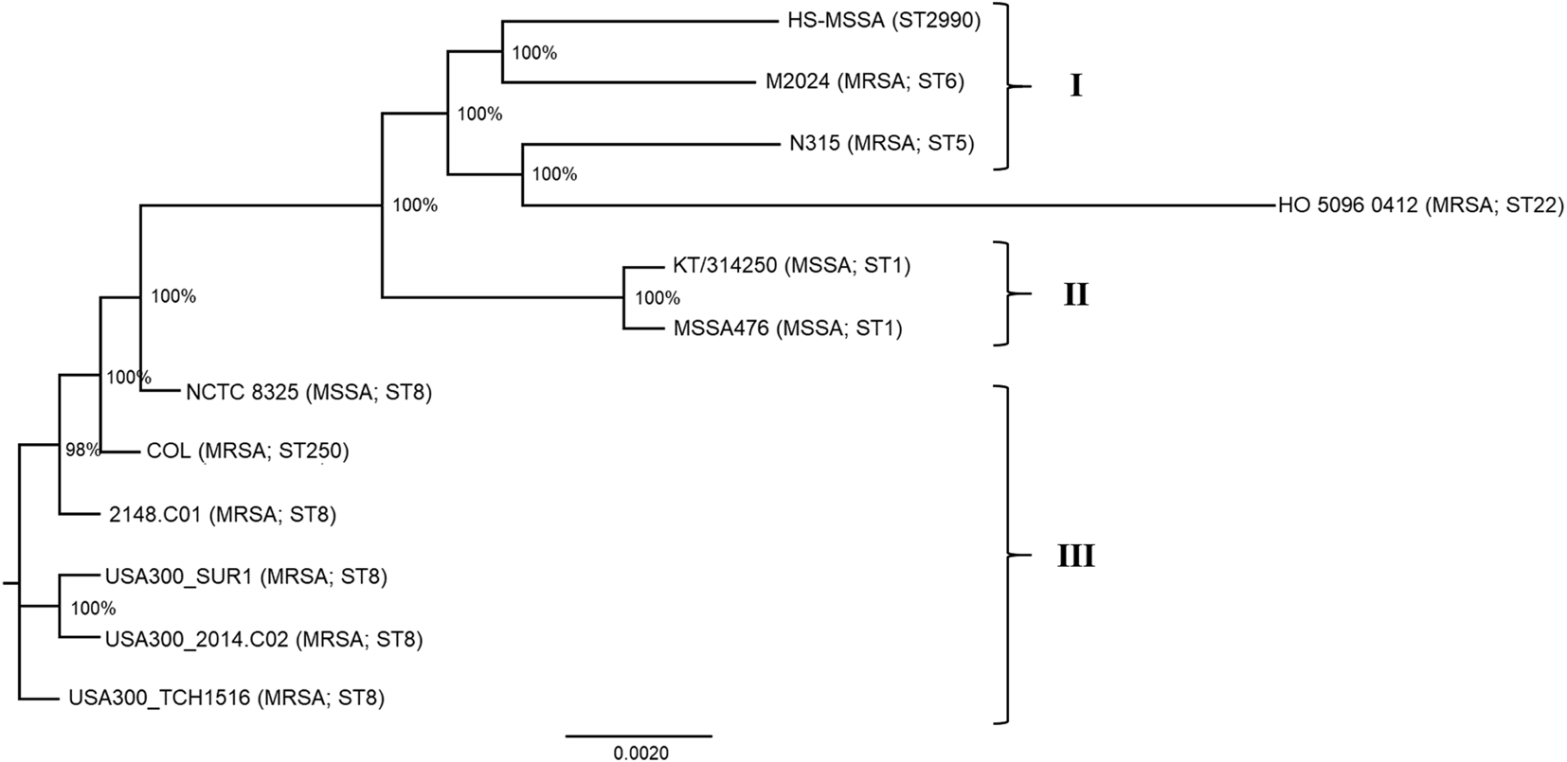
Phylogenomic tree inferred by maximum-likelihood method from SNP-based core genomes alignment of the *S. aureus* strains. The multiple genome sequence alignments of 11 global *S. aureus* strains were generated by the online tool REALPHY using *S. aureus* NCTC 8325 as the reference genome. An unrooted phylogenomic tree was inferred by approximately Maximum Likelihood method using Generalized Time-Reversible (GTR) model with gamma distribution of rates and 100 bootstrap replicates. Bootstrap support value is indicated as a percentage at each node. The roman numerals denote the arbitrary clusters of strains. The ST22 reference genome HO 5096 0412 (NC_017763) resembles a phylogenetic outgroup.

The phylogenetic analysis results revealed that the HS-MSSA strain formed a distinctive clade by itself, sharing a more recent common ancestry with the MRSA clones in Europe and Japan (M2024 and N315) (Fig. 3). The MRSA strain N315 isolated from the pharyngeal smear of a Japanese patient in 1982 belongs to the clonotype II-A which is prevalent in Japan and USA^67^. Together with another MRSA strain M2024 (NZ_CP047021) which caused recurrent clonal outbreaks in Denmark^49^, the three *S. aureus* strains formed a loose cluster with relatively divergent evolutionary paths and geographical origins (Cluster I). Interestingly, the MSSA476 from the United Kingdom that shares a common clonal complex (CC1) with HS-MSSA formed another distinctive cluster (II) although both are community acquired MSSA capable of causing bacteraemia in human hosts and carried the strain-specific plasmid-borne *bla*Z gene^70^. Similarly, the MSSA strain KT/314250 which was also isolated in Malaysia formed a separate clade distinctive from the HS-MSSA lineage but is tightly clustered with the MSSA476 strain. The representative *S. aureus* strains from Europe (NCTC 8325 and COL) and MRSA clones from America formed a third cluster (III) more distantly related to the HS-MSSA strain.

The phylogenetic relationships of the HS-MSSA strain with the global *S. aureus* strains as predicted by the core genome SNPs analysis supported our inference on the HS-MSSA lineage. The sharing of a relatively recent common ancestry with the European-originated MRSA clone M2024 suggests the existence of an ancestral MSSA strain which, in turn, shares a common ancestry with the MSSA476 lineage in Europe. The descendants of this MSSA lineage had since taken separate evolutionary paths, developing antimicrobial resistance or virulence as an adaptive feature in different settings. The relatively more distant genetic relationship between the HS-MSSA and KT/314250, both isolated in Malaysia, infers that multiple MSSA lineages exist and persisted in this geographical region. This observation is not unexpected, given that previous epidemiological studies in Malaysia often reached the same conclusion that the MSSA populations in Malaysia are genetically diverse^55–57^. Unlike most of the MSSA strains that have circulated in this region, the MSSA strain 128A that shares a common MLST genotype (ST2990) with HS-MSSA only appeared on scientific records in 2014. Based on the limited strain data available in the PubMLST database for the MSSA strain 128A, we hypothesize that the ST2990 strains in Malaysia showed variable clinical manifestations, ranging from non-invasive SSTI to severe bacteraemia. The HS-MSSA lineage has probably accumulated sufficient adaptive features through the acquisition of mobile genetic elements, giving rise to its unusual virulence in causing severe invasive infection in human host^19^. Moreover, the close genetic proximity with the MRSA clones and its genome plasticity also suggests that the HS-MSSA lineage could provide a stable genetic environment to allow the acquisition of genetic elements that confer drug resistance^6^. Therefore, continuous molecular surveillance to detect highly virulent MSSA in clinical settings is essential to allow timely management of invasive staphylococcal infections and the development of potential anti-virulence therapy.

### HS-MSSA (CC1/ST2990) showed a closer phylogenetic relationship with CC5 than CC1 based on cgSNP analysis

**Figure 4:**
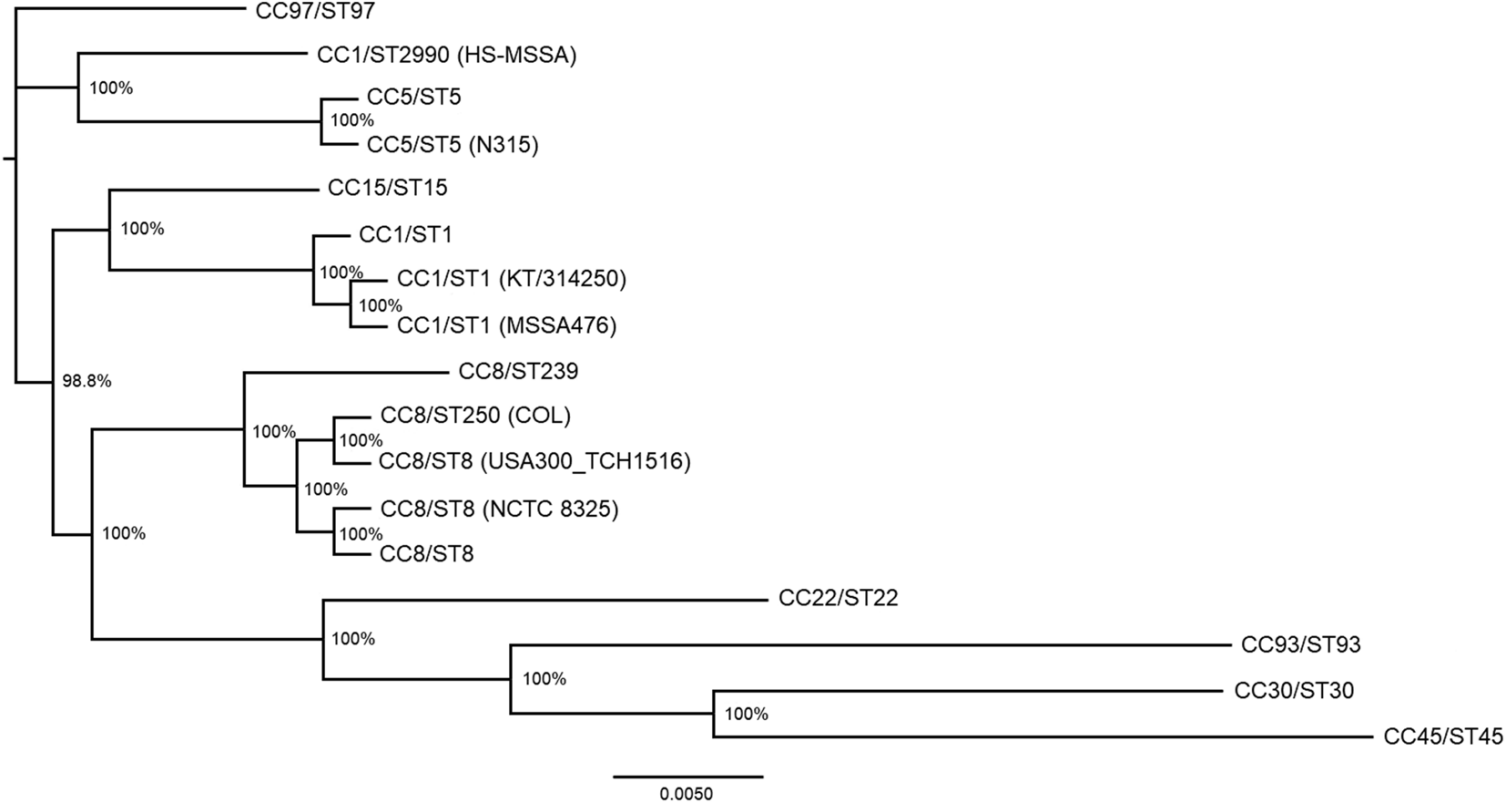
Phylogenomic tree inferred by maximum-likelihood method from SNP-based core genomes alignment of the *S. aureus* strains representing different multi-locus sequence typing (MLST) clonal complexes. The multiple genome sequence alignments of 17 *S. aureus* strains were generated by the online tool REALPHY using *S. aureus* NCTC 8325 as the reference genome. An unrooted phylogenomic tree was inferred by approximately Maximum Likelihood method using Generalized Time-Reversible (GTR) model with gamma distribution rates and 100 bootstrap replicates. Bootstrap support value is indicated as a percentage at each node. The representative genomes for each clonal complex are denoted as clonal complex/sequence type (i.e. CC1/ST1 represents MLST sequence type 1 in clonal complex 1). The PubMLST ID and European Nucleotide Archive (ENA) reference numbers of the representative genomes are available in Supplementary Table S1.

Upon inspection of the MLST clonal complexes of the global *S. aureus* strains selected for cgSNP-based phylogenetic analysis, we observed that the HS-MSSA strain genotyped as CC1/ST2990 was not found on the same clade as other CC1 strains. In order to investigate the accuracy of this phylogenetic clustering, representative genomes of the central STs for the major CCs were retrieved from the PubMLST database for further analysis^71^. Overall, the clustering of the STs corresponded with the CC grouping based on the 7-loci MLST scheme (Fig. 4). However, the HS-MSSA formed a distinctive clade sharing a common node with the CC5/ST5 clade (100% bootstrap support) instead of being grouped in the CC1 cluster. On the contrary, the phylogenetic analysis based on the concatenated sequences of the representative STs revealed that the HS-MSSA indeed showed a closer phylogenetic relationship with CC1/ST1 and CC8/ST8 (92% bootstrap support) (Supplementary Fig. S1). Nonetheless, the multiple genomes alignment of HS-MSSA and the representative CC1/ST1 genomes MSSA476 and ERR410082 (PubMLST ID: 5965) showed that multiple genomic insertions were observed in both variable (PR1-PR3) and conserved regions of the HS-MSSA genome (Supplementary Fig. S2). This could have contributed to the greater phylogenetic divergent of HS-MSSA from other CC1 strains based on cgSNP analysis.

Our observations support the previous notion that the current grouping of *S. aureus* strains into clonal complexes based on the 7-loci MLST scheme requires reconsideration to better discriminate the strains^71^. This observation is not unexpected as cgSNP-based analysis infers the phylogenetic relationship between organisms based on thousands of core genes, hence providing a greater resolution than the 7-loci MLST that examines a relatively small portion of the genome. Nonetheless, current advancement in next-generation sequencing technology has improved the resolution of MLST-based phylogeny. The core genome MLST (cgMLST) which involves the assessment of 1351 loci (for *S. aureus*) and the whole genome MLST (wgMLST) are the more appropriate approaches for inferring phylogenetic relationship of genetically clonal strains. Previous studies using different bacterial species have observed a high concordance between cgMLST and wgMLST with SNP-based analysis, generating phylogenetic trees with great statistical similarity and almost equivalent clustering of bacterial strains^72,73^. Therefore, the current clustering of ST2990 into CC1 needs to be re-assessed by the scientific community. Nonetheless, more ST2990 genomes should be included for cgSNP-based analysis in the future to verify this notion. The present study is limited by only one ST2990 (HS-MSSA) genome that was available on publicly accessible genomic databases at the time of writing.

## Conclusion

We report herein a novel genotype of MSSA (t091/ST2990) of community origin that caused severe sepsis in the human host. Upon examination, the HS-MSSA genome harbours only very few AMR genes, which was reflected in its pan-susceptible phenotype. The HS-MSSA strain carries multiple virulence genes in its genome, including prominent staphylococcal toxin genes that are associated with mobile genetic elements (prophages and SaPI). The HS-MSSA strain shares a common ancestry with the MRSA clone originated from the European region, but instead of developing drug resistance, it has evolved through a different path via the acquisition of multiple virulence and pathogenicity factors. Due to the limited epidemiological data available for this novel genotype (t091/ST2990), we could not conclude that this MSSA lineage has emerged locally or the HS-MSSA strain was just a sporadic strain imported from other regions without molecular typing data. Although the patient reported no history of recent travels upon admission, we cannot rule out the possibility of local transmission. Therefore, increased effort in molecular surveillance is essential to allow the development of appropriate treatment strategies to reduce the mortality risk of severe invasive infections caused by this organism.

## Supplementary Information

Supplementary Information 1.

Supplementary Information 2.

Supplementary Information 3.

Supplementary Information 4.

## Data Availability

All data generated or analysed during this study are included in this published article. The genomic data of the HS-MSSA strain generated and analysed during the current study are available in the NCBI GenBank database at https://www.ncbi.nlm.nih.gov/genome/, under the accession VCMW00000000.
