# Supplementary figures and images for: Genomic analysis revealed a novel genotype of methicillin-susceptible Staphylococcus aureus isolated from a fatal sepsis case in dengue patient
Source: Sci Rep. 2021 Mar 1;11:4228. doi: 10.1038/s41598-021-83661-8 (PMC7921411; doi:10.1038/s41598-021-83661-8)

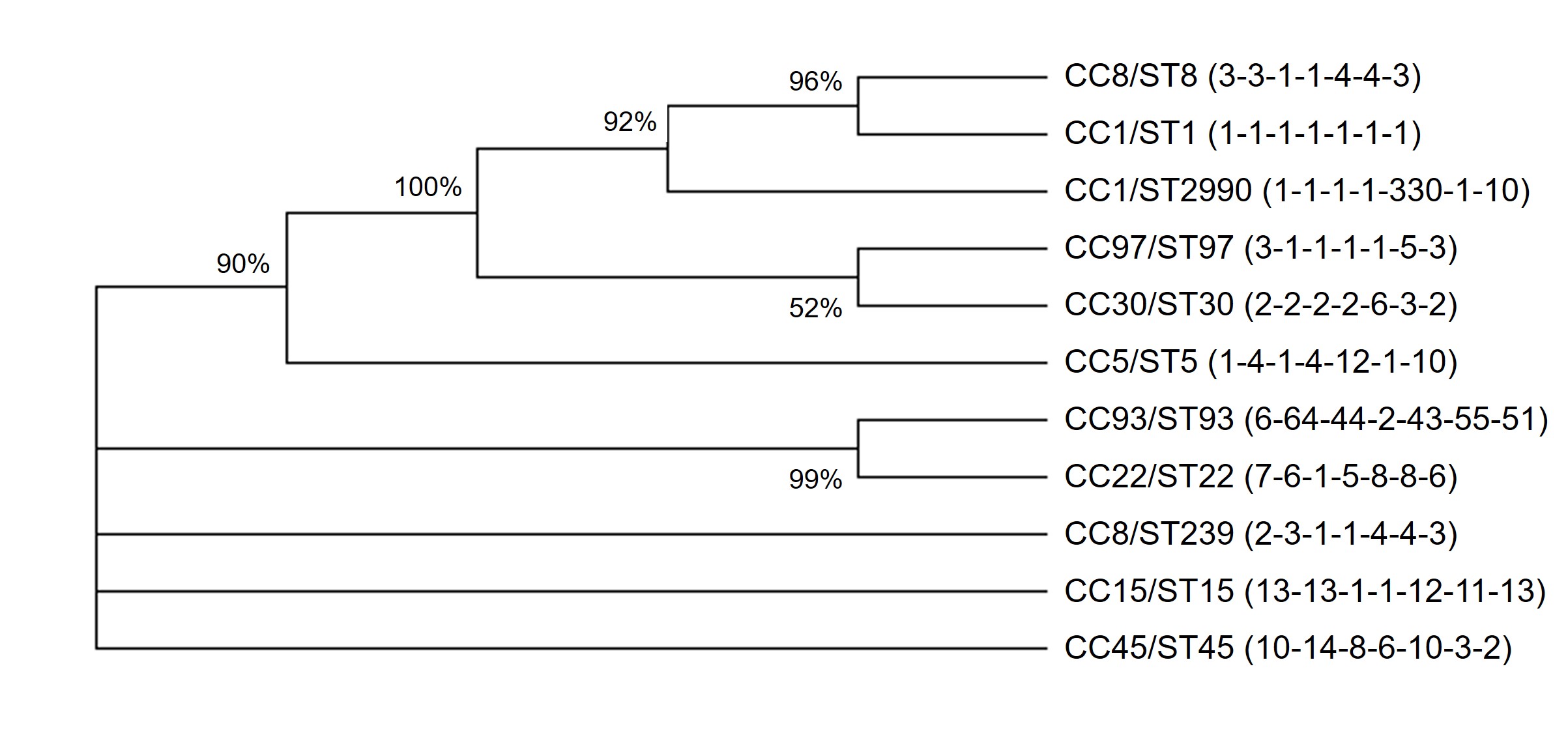

Supplement: Supplementary file 1 — Supplementary Information 1. [file 41598_2021_83661_MOESM1_ESM.jpg]

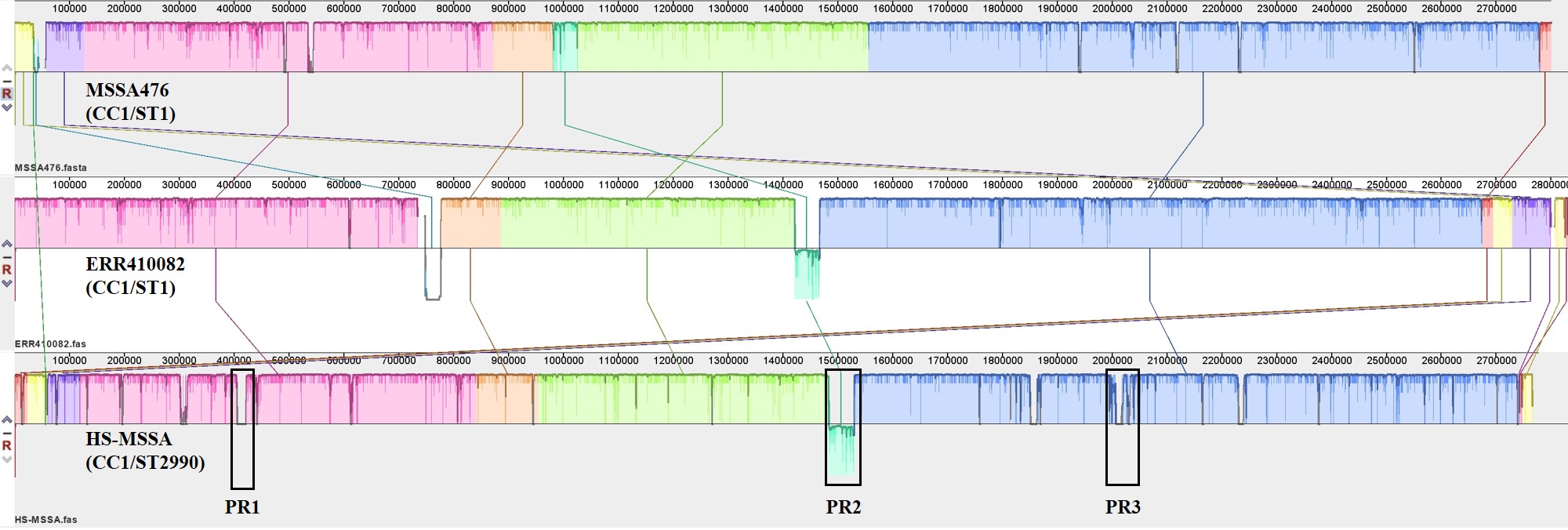

Supplement: Supplementary file 2 — Supplementary Information 2. [file 41598_2021_83661_MOESM2_ESM.jpg]
