# Supplementary material for: Genomic analysis revealed a novel genotype of methicillin-susceptible Staphylococcus aureus isolated from a fatal sepsis case in dengue patient
Source: Sci Rep. 2021 Mar 1;11:4228. doi: 10.1038/s41598-021-83661-8 (PMC7921411; doi:10.1038/s41598-021-83661-8)
Supplement: Supplementary file 4 — Supplementary Information 4. [file 41598_2021_83661_MOESM4_ESM.docx]

**Supplementary Figure S1** Phylogenetic tree of the major MLST clonal complexes based on concatenated allelic sequences. The evolutionary history was inferred by using the Maximum Likelihood method and General Time-Reversible model with 1000 bootstrap replicates. Bootstrap support value is indicated as a percentage at each node. The rate variation model allowed for some sites to be evolutionarily invariable ([+I], 47.47% sites). The allelic profile of each sequence type is represented as a string of numbers arranged in the order arcC-aroE-glpF-gmk-pta-tpi-yqiL.

**Supplementary Figure S2** The genomic alignment of HS-MSSA chromosome against the genomes of CC1/ST1 strains. The similarity plot was generated using Mauve version 2.4.021. Genomic regions with a low percentage of similarity are seen as gaps in the similarity plot of the respective genomes. The relative positions of the phage-associated regions (denoted as PR1 to PR3) on the HS-MSSA chromosome are highlighted in black boxes.
